# Metastasis-associated fibroblasts promote angiogenesis in metastasized pancreatic cancer via the CXCL8 and the CCL2 axes

**DOI:** 10.1038/s41598-020-62416-x

**Published:** 2020-03-25

**Authors:** Thomas M. Pausch, Elisa Aue, Naita M. Wirsik, Aida Freire Valls, Ying Shen, Praveen Radhakrishnan, Thilo Hackert, Martin Schneider, Thomas Schmidt

**Affiliations:** Department of General, Visceral and Transplantation Surgery, Heidelberg University Hospital, Heidelberg, Germany

**Keywords:** Pancreatic cancer, Preclinical research, Surgical oncology

## Abstract

The characteristic desmoplastic stroma of pancreatic ductal adenocarcinoma (PDAC) is a key contributor to its lethality. This stromal microenvironment is populated by cancer-associated fibroblasts (CAFs) that interact with cancer cells to drive progression and chemo-resistance. Research has focused on CAFs in the primary tumour but not in metastases, calling into question the role of analogous metastasis-associated fibroblasts (MAFs). We infer a role of MAFs in murine hepatic metastases following untargeted treatment with the anti-angiogenic drug sunitinib *in vivo*. Treated metastases were smaller and had fewer stromal cells, but were able to maintain angiogenesis and metastasis formation in the liver. Furthermore, sunitinib was ineffective at reducing MAFs alongside other stromal cells. We speculate that cancer cells interact with MAFs to maintain angiogenesis and tumour progression. Thus, we tested interactions between metastatic pancreatic cancer cells and fibroblasts using *in vitro* co-culture systems. Co-cultures enhanced fibroblast proliferation and induced angiogenesis. We identify carcinoma-educated fibroblasts as the source of angiogenesis via secretions of CXCL8 (aka IL-8) and CCL2 (aka MCP-1). Overall, we demonstrate that metastasis-associated fibroblasts have potential as a therapeutic target and highlight the CXCL8 and CCL2 axes for further investigation.

## Introduction

Ductal adenocarcinoma of the pancreas (PDAC) is an aggressive cancer with slim chances of survival^1–3^. Once metastasized, chemotherapy provides the main treatment option but standard regimes offer minimal survival extension^4–6^. PDAC’s chemo-resistance may involve the characteristic desmoplastic stroma that comprises most of the tumour tissue^7–11^. The stroma contains a population of carcinoma-associated fibroblasts (CAFs) that can differentiate from pancreatic stellate cells, among other sources^12,13^. CAFs surround cancer cells and provide structural and signalling functions^9,13–21^. Thus, the mechanisms that activate stromal fibroblasts during cancer progression have potential as therapeutic targets^12,13,22–24^.

CAFs are a target for novel PDAC therapies, but there is controversy over their role in tumour progression^13^. On one hand, stromal depletion from PDAC-like tumours using Hedgehog (Hh) pathway inhibitors can stimulate angiogenesis and enhance drug delivery^25^. On the other, stromal myofibroblast depletion can suppress angiogenesis and enhance tumour progression^26–28^. Together, these studies imply that the stroma can have both a protective role for the tumour^28^ but also restrict its progression^27,29^. Importantly, the balance between these roles may depend on sub-populations of different fibroblast types in the microenvironment^22,30^.

Clearly, improved treatment requires a better understanding of the stroma and its fibroblastic populations. However, even less is known about cancer-fibroblast interactions during metastasis, despite the largely stromatic composition of metastatic tissue^7–11,22,23,31,32^. Metastasis-associated fibroblasts (MAFs) may be analogous to CAFs in the primary tumour microenvironment, and may also be largely sourced from stellate cells at the site of metastasis. But their roles and origins have yet to be fully illustrated^22,31^.

Thus, we aim to investigate the supportive role of cancer-associated fibroblasts with an *in vivo* murine model of developed hepatic metastases subjected to the angiogenesis inhibitor sunitinib. We follow with an *in vitro* experiment that intends to provide a generalized model of *in vivo* crosstalk between cancer cells and fibroblasts in the metastatic PDAC microenvironment. We specifically consider the consequences of this interaction on cell proliferation and angiogenesis.

## Results

### Sunitinib reduces metastatic tumour size and volume *in vivo*

We injected mice in the portal vein with highly aggressive Panc02^33^ cancer cells, isolated from metastasis, and allowed eight days for establishment (Fig. S1). Mice were treated for a further eight days with the drug sunitinib, an anti-angiogenic, tyrosine kinase inhibitor (Supplementary Methods). Sunitinib has a high affinity to VEGF and PDGF receptors, and thereby also targets CAFs. We sacrificed the mice at day 17 and compared the metastasized livers of sunitinib-treated and untreated control mice.

**Figure 1:**
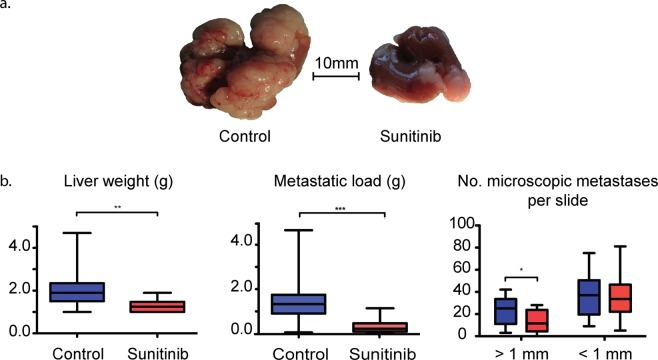
Effects of short-term sunitinib administration on metastasised liver condition *in vivo*. (**a**) Representative images of livers from control and sunitinib treated mice (images have been modified to remove the background), and (**b**) comparisons of liver weight, metastatic load (liver weight * percentage volume metastasized), and number of micrometastases per slide between control (blue) and sunitinib treated (red) mice. Significance values from a two-tailed Student’s t-test: *p < 0.05; **p < 0.01; ***p < 0.001. Photo credits TP and EA.

Injecting Panc02 cancer cells through the portal vein generated extensive metastasis throughout the liver. However, mice treated with sunitinib showed up to 80% reduced metastatic load (liver weight * percentage metastasised) compared to the diffuse metastases of control mice (Fig. 1a, Table S2). The livers of treated mice were significantly lighter, with significantly fewer metastases larger than 1 mm (Fig. 1b). However, metastases smaller than 1 mm were not significantly affected by sunitinib.

### Sunitinib diminishes stromal cells but not activated myofibroblasts *in vivo*

**Figure 2:**
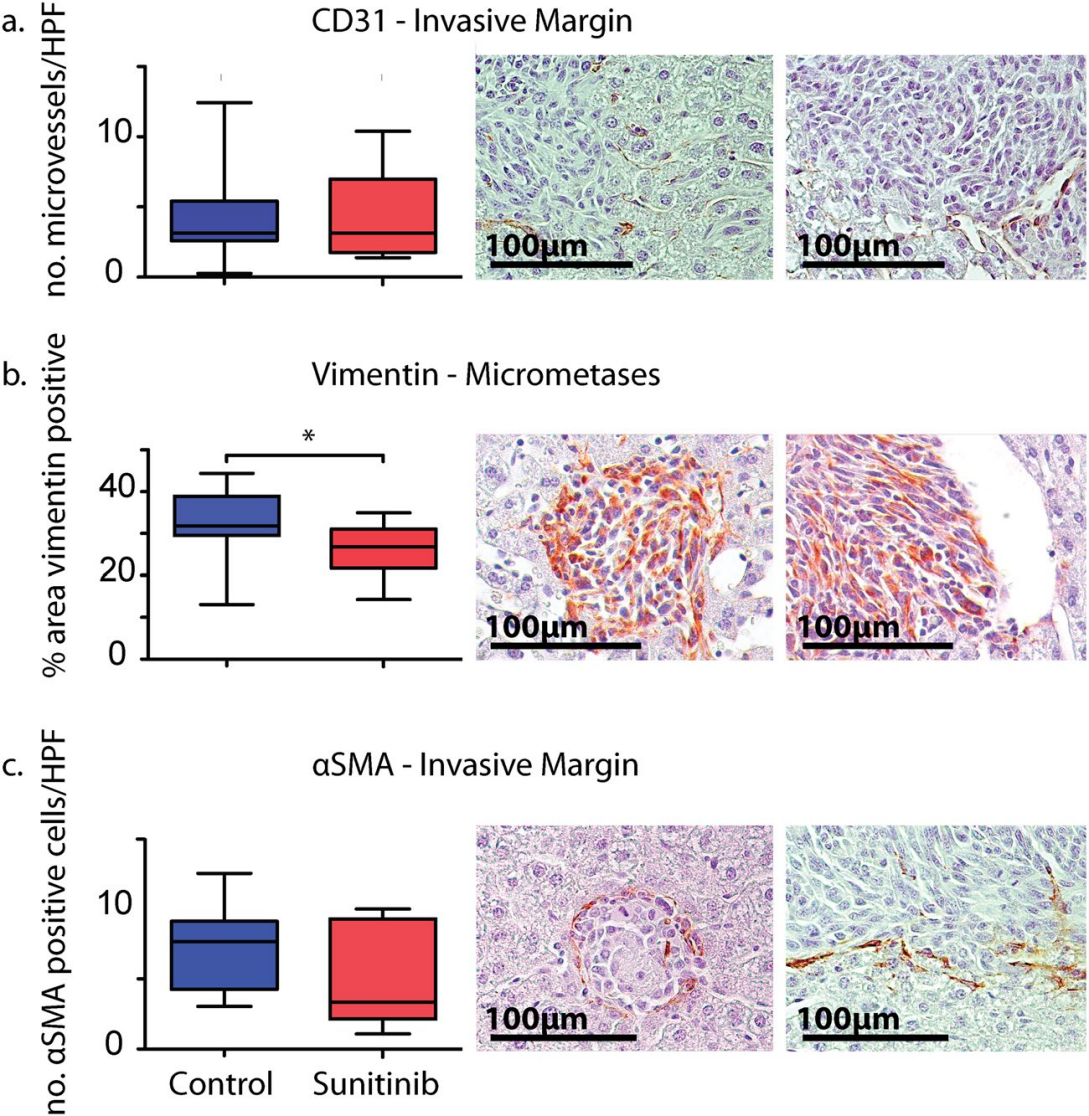
Immunohistochemical analysis of tumour cell proliferation and composition in liver metastases. Comparisons of (**a**) microvessel density (CD31), (**b**) mesenchymal cells (vimentin), and (**c**) activated MAFs (α-SMA) between control (blue) and sunitinib treated (red) mice. Also shown are representative images of sections from control (left) and treated (right) mice. Significance values from a two-tailed Student’s t-test: *p < 0.05; **p < 0.01; ***p < 0.001 (Table S2). All photo credits by TP and EA.

**Figure 3:**
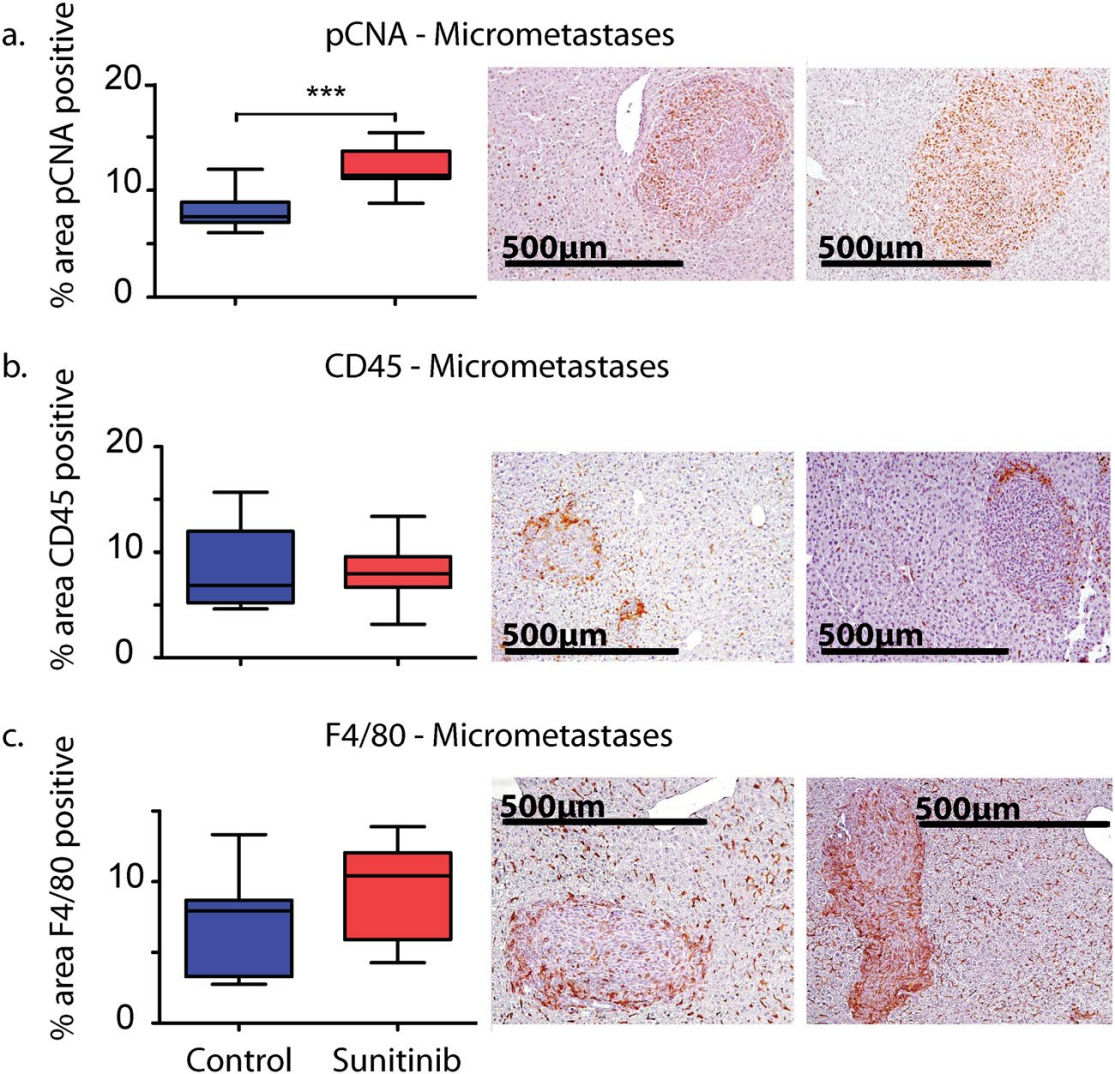
Immunohistochemical analysis of tumour cell proliferation and composition in liver metastases. Comparisons of (**a**) cell proliferation (pCNA), (**b**) lymphocytes (CD45), and (**c**) macrophages (F4/80) between control (blue) and sunitinib treated (red) mice. Also shown are representative images of sections from control (left) and treated (right) mice. Significance values from a two-tailed Student’s t-test: *p < 0.05; **p < 0.01; ***p < 0.001 (Table S2). All photo credits by TP and EA.

We further investigated the effects of sunitinib on the tumour microenvironment with immunohistochemical assays of tumour proliferation and cell-composition (Figs. 2 and 3, Table S2). We focus on areas of tumour growth at the invasive front and micrometastases. Firstly, sunitinib treatment did not significantly affect microvessel density at the invasive front (CD31; Fig. 2a). Secondly, while treated mice had significantly fewer mesenchymal cells within the metastatic lesion (vimentin; Fig. 2b), the invasive margin did not show any significant difference in the number of activated MAFs (α-SMA; Fig. 2c). Thirdly, markers for cell proliferation (pCNA) were significantly greater in micrometastases of sunitinib-treated tumours (Fig. 3a). Finally, there were no significant differences in lymphocytes (CD45; Fig. 3b) or macrophages (F4/80; Fig. 3c) between treated and untreated micrometastases. Taken together, sunitinib appears to be effective at reducing metastasis size, but not at reducing angiogenesis and activated MAFs at the invasive margin. Therefore, we follow with *in vitro* experiments that focus on the angiogenic consequences of crosstalk between cancer cells and fibroblasts.

### Pancreatic cancer cells stimulate proliferation of normal human fibroblasts

We first assayed proliferation in fibroblast and cancer cells that had been exposed to each other. We cultured normal human dermal fibroblasts (NHDF), the metastatic pancreatic cancer line T3M4, and co-cultures of both cell types (Fig. S2). We cultured NHDF cells in a starving medium, a T3M4 medium, or a medium of co-cultured cells. The medium had a significant effect on proliferation in NHDF cells (F_3, 40_ = 6.66, p = 0.001, r^2^ = 0.33, Fig. S3a), as NHDF grown in T3M4 proliferate significantly more than in starving medium (q_s_ = 5.82, p < 0.010). We also cultured T3M4 cells across mediums but found consistent proliferation (F_3, 40_ = 0.63, p = 0.603, r^2^ = 0.04, Fig. S3b).

### Cancer-educated fibroblasts enhance angiogenesis

We continued by measuring the formation of blood vessels in human umbilical cord endothelial cells (HUVECs) that had been exposed to NHDF, T3M4 or co-culture. We measured the number of branches and total length of the tube network, and normalized these values to a hypothetical effect. Specifically, the hypothetical effect size of one is the mean difference between HUVEC tube networks conditioned with starving medium (negative control) and vascular endothelial growth factor, VEGF (positive control).

**Figure 4:**
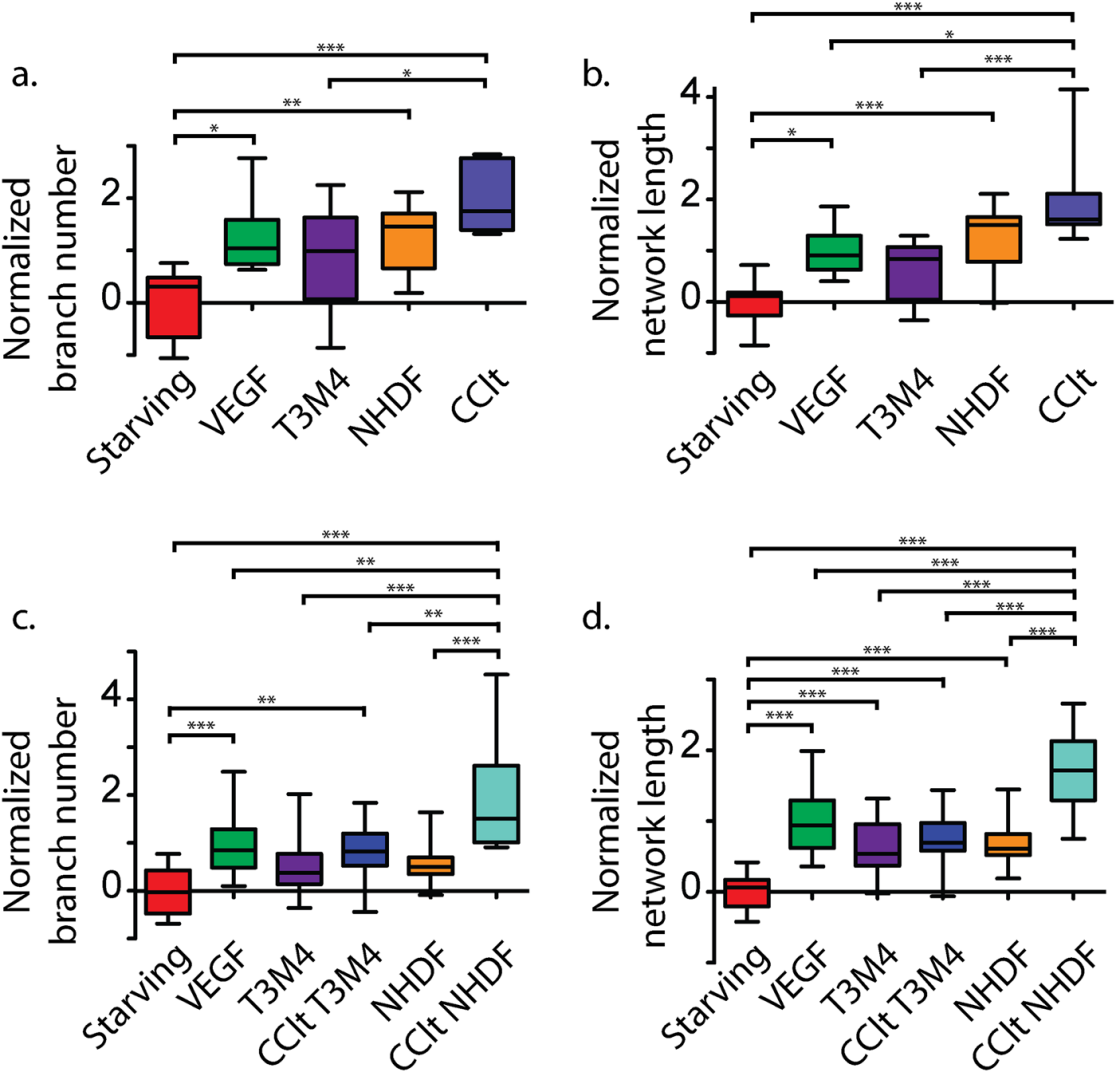
HUVECs tube formation in conditioned media. Number of network branches (**a**,**c**) and total tube length (**b**,**d**) in HUVECs exposed to conditioned media in normal (**a**,**b**) and extended (**c**,**d**) treatments: negative control (Starving), positive control (VEGF), singular T3M4, singular NHDF, T3M4-NHDF co-culture (CClt), co-cultured T3M4 (CClt T3M4), co-cultured NHDF (CClt NHDF). All measurements are normalized to the distance between control means. Experiments were performed in triplicate and analysed with ANOVA, using Tukey’s test for pair-wise comparisons: *p < 0.05; **p < 0.01; ***p < 0.001.

The normalized number (F_4, 39_ = 8.86, p < 0.001, r^2^ = 0.48) and total length (F_4, 40_ = 12.32, p < 0.001, r^2^ = 0.55) of branches that grew in HUVECs depended on the conditioning medium (Fig. 4a,b). According to normalized means, HUVECs conditioned with T3M4-NHDF co-culture had around twice the angiogenic effect that VEGF had over starving medium. A post-hoc Tukey’s comparison indicates that co-culture promoted significantly more tubes (q_s_ = 8.17, p < 0.001) than the starving control, and significantly longer networks than the VEGF control (q_s_ = 4.60, p < 0.050). On their own, NHDF cells promoted about 120–130% as much angiogenesis as VEGF, which represents a significant difference to the starving control (tube number: q_s_ = 4.96, p < 0.010; total length: q_s_ = 6.08, p < 0.001) but not to the VEGF control (tube number: q_s_ = 0.12, p > 0.050; total length: q_s_ = 1.23, p > 0.050). On the other hand, HUVECs cultured with T3M4 cancer cells did not show any significant angiogenic difference to the starvation medium (tube number: q_s_ = 3.29, p > 0.050; total length: q_s_ = 3.06, p > 0.050).

We sequentially conditioned the media to establish an extended co-culture protocol, allowing us to distinguish the independent effects of co-cultured cancer and fibroblast cells (Fig. 4c,d). There were significant differences in the normalized tube number (F_5, 84_ = 12.94, p < 0.001, r^2^ = 0.44) and total tube length (F_5, 84_ = 29.68, p < 0.001, r^2^ = 0.64) between conditioning media. Specifically, angiogenic effects were close to double the VEGF control when HUVECs were conditioned with co-cultured fibroblasts. Tukey’s tests demonstrate that fibroblasts educated in a medium of cancer cells promoted greater angiogenesis than non-educated NHDF (number: q_s_ = 7.01, p < 0.001; length: q_s_ = 9.65, p < 0.001). By contrast, T3M4 cells from co-culture promoted slightly less angiogenesis than the VEGF control, and were non-significantly elevated compared to non-co-cultured T3M4 (number: q_s_ = 2.17, p > 0.050; length: q_s_ = 1.01, p > 0.050).

### Fibroblasts upregulate the pro-angiogenic factors CXCL8 and CCL2 in co-culture

**Figure 5:**
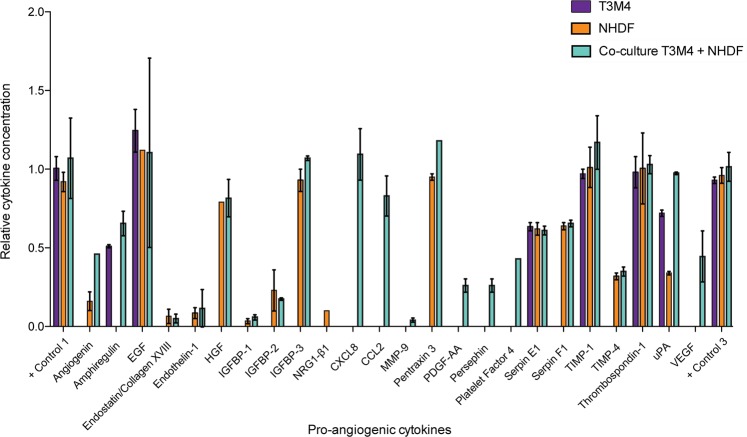
Pro-angiogenic cytokine concentrations across conditioned media. Relative mean cytokine concentration ± SD (based on pixel density normalized to positive spot pixels) for pro-angiogenic cytokines expressed by T3M4, NHDF and T3M4-NHDF co-culture.

We analysed the proteomes of conditioned media to identify the key pro-angiogenic factors in co-culture (Fig. 5). This assay indicated the exclusive expression of monocyte chemotactic protein 1 (CCL2, aka MCP-1**)** and interleukin 8 (CXCL8, aka IL-8) in co-cultured media. We used qPCR to determine the source of secretions (Fig. S4), and found that the expression of both CCL2 (t_8.69_ = 11.98, p < 0.001) and CXCL8 (t_8_ = 4.39, p = 0.002) were elevated only in fibroblasts.

### CCL2 and CXCL8 induce angiogenesis

**Figure 6:**
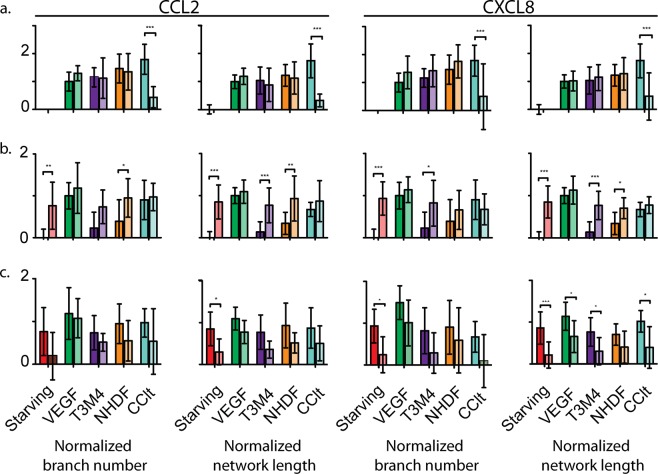
Effects of CCL2 and CXCL8 neutralization on HUVEC tube formation. Normalized mean ± SD number of network branches and total tube length in HUVECs when (**a**) antibodies added to normal conditioned media (normal on the left, antibodies on the right), (**b**) recombinant proteins added to normal conditioned media (normal on the left, recombinant on the right), and (**c**) antibodies added to recombinant proteins (recombinant on the left, antibodies on the right). Values are normalized to the mean distance between controls. Experiments were performed in triplicate and analysed with ANOVA. Within-treatment comparisons represent single effects ANOVAs for (**a**) natural proteins and (**b**,**c**) Bonferroni posthoc tests for recombinant proteins: *p < 0.05; **p < 0.01; ***p < 0.001.

We confirm the angiogenic functions of CCL2 and CXCL8 in co-culture by incubating conditioned media with antibodies that block them. Antibodies (for both proteins) only significantly inhibited angiogenesis in HUVECs conditioned with co-culture (Fig. 6a; Tables S3–S10). HUVEC tube formation in co-cultures was also significantly inhibited by neutralizing the CXCL8 receptors, CXCR1 (aka IL-8-Rα, Table S9) and CXCR2 (aka IL-8-Rβ, Table S10).

To confirm our findings we repeated the above experiment using recombinant CCL2 and CXCL8 (Fig. 6b, Tables S11–S14). We observed a significant enhancing effect of adding recombinant proteins on HUVEC angiogenesis in both T3M4 and NHDF (only for length). However, there was no significant effect of recombinant proteins on co-cultured media. This is likely due to the saturation of receptors with intrinsic proteins (see Supplemental Discussion). Adding neutralizing antibodies to recombinant proteins significantly inhibited angiogenesis across all treatments for both CCL2 and CXCL8 (Fig. 6c, Tables S15 and S16).

## Discussion

Our *in vivo* study demonstrates the consequences of untargeted sunitinib treatment on the tumour microenvironment. Sunitinib significantly diminished mesenchymal cells and tumour size, but was ineffective at inhibiting angiogenesis. Furthermore, the proliferation of cancer cells was greatest at invasion sites in treated livers. These results are consistent with the model of stroma acting as a barrier against drug delivery, but also as a restriction on cancer growth^25,27,28,34–36^. Thus, sunitinib may have released cancer cells from stromal suppression to result in substantially smaller, but more aggressive metastatic tumours. Furthermore, depletion of the desmoplastic stroma did not include activated myofibroblasts at the invasive tumour margin. This implies that the ratio of cells in the tumour microenvironment shifted to the more pro-tumorigenic myofibroblasts (i.e. MAFs), and their potential to promote cancer proliferation and sunitinib resistance^13,22,24,31^.

Our findings speak to the complex interactions between tumours and their microenvironment^1^. Sunitinib treated mice expressed a reduction in vimentin-positive mesenchymal cells, which comprise much of the desmoplastic stroma^32,37^. Hence, the overall tumor burden (which is calculated from liver weight and metastasized volume) may be reduced from stromal depletion. However, the proportion of αSMA-positive activated myofibroblasts were not reduced by sunitinib treatment. In contrast to other mesenchymal cells, these cells may promote the proliferation of cancer cells in hepatic metastases of pancreatic cancer^38^ and many other solid tumors^39^.

The tumor-promoting properties of activated myofibroblasts are implied by studies that demonstrate the failure of antiangiogenic therapies that target VEGF-pathways of metastatic tumors, despite their effectiveness against primary tumors^40,41^. In these studies, antiangiogenic therapy may accelerate invasiveness and metastasis in micro-metastatic and early stages, especially following short-term treatment^40^. These failures may arise due to acquired resistance in the tumour, such as the induction of alternative pro-angiogenic pathways (e.g. overexpression of CXCL8 or FGF^42–44^). Alternatively, antiangiogenic therapies may fail for metastatic tumours due to tumour-independent (host-mediated) resistance^29,40^. Thus, the metastatic cells of sunitinib treated mice in our study may similarly be resistant against antiangiogenic therapy, allowing them to proliferate and spread.

The complexity of the tumor-microenvironment has serious therapeutic implications, as evidenced by conflicting outcomes for therapies that target the stroma and/or fibroblast populations. For instance, therapies that target stromal depletion by inhibiting the Hedgehog-signaling (Hh) pathway in primary tumors can improve patient survival by reducing tumor burden and metastases^25,36^. These results may relate to a reduction in the chemo-resistance of a hypovascular and hypoxic stromal environment that reduces concentrations of chemotherapeutic agents^25,45,46^ and activated myofibroblasts that uptake and store them^35^. However, later studies that use Hh-pathway inhibition or targeted depletion of αSMA-positive myofibroblasts can result in tumor cell proliferation and other detrimental effects^27,28^. These later studies imply that the stroma may also function to suppress the proliferation of tumor cells. Importantly for PDAC, activated myofibroblasts and desmoplastic stroma (which comprises collagen and fibroblasts) do not necessarily vary together^47^. For instance, cancer-associated fibroblast populations can be high relative to collagen depositions in the peritumoral invasion zone during early disease, but relatively low in established desmoplastic regions. Taken together, there is a complex web of interdependencies and effects between the stroma, fibroblast populations, immune cells and cancer cells. Thus, effective therapy requires anti-desmoplastic or anti-angiogenic treatments that provoke changes in the tumor microenvironment that disadvantage the tumor, and care must be taken to avoid supporting the tumor.

Accordingly, we used media from co-cultures of normal fibroblasts and metastatic pancreatic cancer cells to examine cellular proliferation and angiogenesis *in vitro*. Our experiments demonstrate that cancer cells stimulate the proliferation of fibroblasts and educate them to express pro-angiogenic proteins CXCL8 and CCL2. These results may translate to human PDAC *in vivo*, in which CAFs densely surround and interact with cancer cells to play pivotal roles in tumour progression^15–17^. However, this experiment is only the first step to understand the consequences of crosstalk between metastatic cancer cells and fibroblasts at an invasion site. Specifically, we only considered T3M4 and NHDF cells as a generalized model of PDAC-fibroblast interactions, whereas we could achieve a more complete understanding using also wild-type and mutant cancer cells and fibroblasts from multiple origins. Hence, we will add to the breadth of our findings by repeating the *in vitro* study with patient-derived cell lines and fibroblasts extracted from fresh resections. Our efforts will be supported by an immunohistological screening of the proteins in tissue banks of healthy, cancerous and inflamed pancreata.

We identify the proteins CXCL8 and CCL2 as pro-angiogenic agents in the cancer-fibroblast co-culture, just as they are known to be in other PDAC microenvironments (see also Supplemental Discussion)^23,24,48^. CXCL8 and its receptors CXCR1 and CXCR2 are implicated in the progression of pancreatic cancer^49,50^, and the expression of CXCL8 has a negative prognostic correlation^29,51,52^. CXCL8 is a major pro-angiogenic factor^53–57^, functioning through its receptor CXCR2^58,59^. In addition to its angiogenic functions, CXCL8 is also involved with maintaining hepatic metastatic lesions in an occult form, so long as the liver is uninflamed and hepatic stellate cells are not differentiating into MAFs^31^. Overall, the CXCL8 axis is a therapeutic target in PDAC and blockading the axis reduces pancreatic cancer stem cells, invasion and metastases^51^. The chemokine CCL2 is a chemo-attractant involved with managing cell movement in an immune response. CCL2 can be highly expressed in PDAC tumours and cell lines^60,61^ and has been shown to affect tumour growth and metastases^62,63^, also in urinary bladder cancer^24^. Metastatic PDAC requires CCL2 for immune suppression, and the chemokine is thus highlighted as a therapeutic target^23^.

Together our experiments elucidate therapeutically relevant interactions in the metastatic PDAC microenvironment^7,9,23,30,64^. Some therapeutic approaches consider manipulating the microenvironment’s vasculature to either starve the tumour (by decreasing perfusion) or enhance drug delivery (by increasing perfusion)^7,9,65^. Our *in vitro* study demonstrates that metastatic cancer cells can educate normal fibroblasts to secrete pro-angiogenic proteins (that can be neutralized). Thus, upregulating these proteins may allow chemotherapeutic compounds to bypass the stromatic barrier. Alternatively, inhibiting these proteins may serve as a more effective therapy for suppressing vascularization in metastatic tumours than untargeted sunitinib treatment. Other therapeutic approaches consider manipulating the functions of the fibroblast populations^13,22,23,31^. For instance, upregulating CXCL8 can interfere with the activation of metastasis-associated fibroblasts by keeping precursor stellate cells in a quiescent state^31^. Alternatively, inhibiting CCL2 secretion may weaken the immunosuppressive phase of hepatic metastasis^23,24^.

The tumour microenvironment is a complex system of dynamic interdependencies. Fibroblasts and related cells are integral to PDAC’s aggression and resistance, and are keys to building successful therapeutic combinations^7,9,12,13,22–24,30,31,64^. Our experiments reveal that active metastatic fibroblasts may aid the formation and colonization of pancreatic cancer by promoting angiogenesis at the boundary of cancer cells and resisting anti-angiogenic compounds like sunitinib.

## Methods

### Untargeted sunitinib treatment of hepatic metastases: *in vivo* experiment

#### Animal model of liver metastasis

Our chosen murine model used artificial seeding of tumour cells instead of real metastatic conditions, but has been validated^66^. All of the animal studies had been governmentally approved according to German regulations of the Animal Welfare Act (TierSchG § 8 Abs.1), Regierungspraesidium Karlsruhe (File G-140/14). We obtained 9–12 week-old female immunocompromised C57BL/6 mice weighing 18–22 g. We performed the studies at the Interfaculty Biomedical Facility of Heidelberg University (IBF, Heidelberg, Germany) according to FELASA and GV-SOLAS guidelines.

Hepatic metastases were induced via portal vein injection of 0.5 × 10^6^ viable cells from sub-confluent cultures of Panc02 (Fig. S1). We used Panc02 due to its highly aggressive nature and establishment as a model of PDAC in a progressed, metastatic stage^67,68^. Cultures were harvested by trypsin treatment and re-suspended as single-cell suspensions in 0.2 mL phosphate-buffered saline (PBS). The control group mice received pure PBS injections.

#### Sunitinib protocol

Hepatic tumours were allowed to metastasize for eight days, post-Panc02 injection (Fig. S1). On day 9, mice were treated with an oral administration of either a) citrate buffer (n = 13), or b) citrate buffer with 40 g sunitinib malate (Supplementary Methods) per kg bodyweight (n = 16). Treatment continued for eight days and mice were euthanized on day 17 to be assessed using stereological and immunohistochemical techniques.

After treating nine mice (control: n = 4, sunitinib: n = 5), we determined an extremely high tumour burden in the control group (70–99% of liver volume) compared to the treatment group (20–60% of liver volume). Thus we changed the protocol for animal welfare compliance to a treatment period of seven days, with euthanasia on day 16. There was no statistical difference in immunohistochemical results between mice from different timelines, so we pool them in all analyses.

#### Stereology

The abdominal cavity was examined for macroscopic hepatic metastases following laparotomy. Livers were excised, weighed, fixed in 10% formalin for 24 hours and then treated with 70% ethanol. Liver sectioning was performed to 4–6 µm thick slices using a microtome and imaged under a microscope with a digital camera (AxioStar Plus microscope with an AxioCam MRc camera). Two independent blinded observers (TP, EA) estimated tumour burden (% volume of the liver with metastases), the number of microscopic metastases (diameter < 1 mm) and macroscopic metastases (diameter > 1 mm). Samples were compared with a two-tailed Student’s t-test or a Welch t-test when variances were unequal.

#### Immunohistochemistry

We prepared tissue sections from formalin-fixed paraffin samples for each animal to gain an overview of metastasis. Samples were deparaffinised, rehydrated, peroxidised (3% peroxide) and then blocked with TNB Blocking Buffer (containing TSA Blocking Reagent) for 30 min at room temperature. We also used hemalaun stainings with eosin counterstainings on some slides after being deparaffinised in order to complete our overview of metastasis (Fig. S5). Tissue sections were processed for heat-induced antigen retrieval.

We applied antigens in blocking buffer to assess tumour growth and composition. We aimed to quantify: i) microvessel density (dilution 1:200 rabbit anti-mouse CD31), ii) mesenchymal cells (1:200 vimentin), iii) activated MAFs (1:200 α-SMA), iv) cell proliferation (1:400 pCNA), v) lymphocytes (1:400 CD45), vi) macrophages (1:200 F4/80). Negative controls were section stained only with the corresponding secondary antibodies, applied at dilutions of 1:400, to rule out non-specific binding (Fig. S6).

Primary antibodies were incubated for 60 minutes at room temperature, followed by washes in blocking buffer. Sections were then incubated with horseradish peroxidase and conjugated secondary antibody. We stained with diaminobenzene (DAB) and counterstained with hemalaun. Imaging was performed using the AxioStar Plus microscope with an AxioCam MRc camera. We analysed randomly chosen high power fields (HPF) of micrometastases or the invasive margin. Two independent blinded observers used ImageJ to evaluate samples for DAB-positive area (for vimentin, pCNA, CD45, F4/80), DAB-positive microvessels per HPF (mean number in 20 HPF for CD31) or DAB-positive cells per HPF (mean number in 20 HPF for α-SMA). Cell clusters of at least one endothelial cell were defined as microvessels when evaluating CD31 (lumen and blood cells not required)^69–71^. Treated and untreated samples for all stainings were compared using a two-tailed Student’s t-test.

#### Animal welfare

All of the animal studies were approved according to German regulations of the Animal Welfare Act (TierSchG § 8 Abs.1, see http://www.ak-tierschutzbeauftragte.berlin/empfehlungen), Regierungspraesidium Karlsruhe (File G-140/14). Post tumour induction, mice were clinically evaluated twice for specific clinical symptoms. Analgesia was administered via a subcutaneous injection of 0.3 mg buprenorphine per kg bodyweight and post-operatively on clinical signs of pain. Animals showing clinical signs of severe pain, high tumour burden or other neoplasms were immediately sacrificed by cervical dislocation.

### Cell proliferation and angiogenesis in co-culture: *in vitro* experiment

#### Cell lines and culture

We aimed to provide a generalized model of crosstalk between PDAC and fibroblasts. We cultured (Table S1): i) human endothelial cells deriving from umbilical cord veins (HUVECs), ii) a fast-growing human PDAC cell line originating from a lymphatic metastasis of an exocrine pancreatic tumour (T3M4), iii) a normal human dermal fibroblast cell line (NHDF). We also generated specific starving mediums for each cell line. All cell lines were cultured at 37 °C in 95% humidified air containing 5% CO_2_. T3M4 is an established model of an aggressive and pro-angiogenic PDAC in metastatic stages^72^, whilst NHDF serves as a general model of fibroblasts that are susceptible to activation by cancer cells^73^.

#### Co-cultures and collection of conditioned media

Conditioned media were generated from separately seeded T3M4 or NHDF cell cultures, or from co-cultures of both cells. We used ThinCert permeable well inlets containing a 0.4μm polycarbonate membrane to separate T3M4 and NDHF cells. We harvested cells at approximately 70% cell confluence and seeded them into inserts (5 × 10^4^ cells/mL in a volume of 600 µL) and wells (4 × 10^4^ cells/mL in a volume of 1.5 mL). Cells in inserts and wells were separately incubated in their growth media for six hours before they were washed twice with phosphate-buffered saline (PBS). After washing we replaced media with starving media for 18 hours. On day 2 we washed cells then replaced the media with fresh HUVEC starving medium. After incubating for 72 hours, we collected and centrifuged (1000 rpm for 3 min) the conditioned media. We used these co-cultures in tube formation and proteome assays.

We developed an ‘extended’ co-culture protocol to discriminate the additive effects of two cell lines in the same co-culture medium. We incubated co-cultures with T3M4 in inserts and NHDF in wells and vice versa, as described above. We discarded media after 72 hours and washed the cells in inserts twice with PBS. We then separated T3M4 and NHDF by transferring the inserts to fresh wells containing HUVEC starving medium but no cells. After incubating for 48 hours, we collected and centrifuged (1000 rpm for 3 minutes) the conditioned media. Samples not used immediately in experiments were snap-frozen in liquid nitrogen and stored at −80 °C.

#### Proliferation assay

We used the Roche WST-1 cell proliferation reagent to analyse the proliferation of T3M4 (n = 11) and NHDF (n = 11). We seeded 4000 cells/well in 96 well plates and cultured them in cell-specific growth medium until attachment. We then incubated the cultures in starving medium overnight. Consecutively, cultures were transferred to 100 μL of conditioned media (as described above) and incubated for at least six hours until attachment. We replaced conditioned media with fresh media and 10 µL WST-1 in 1:10 concentration. We incubated for 48 hours and measured optical absorbance using Tecan Infinite F200 Pro fluorescent microplate reader (Tecan Life Sciences, Männedorf, Switzerland). Our negative control (effect size = 0) was a background with no cells. We generated positive control media (effect size = 1) by seeding cells in complete medium with 10% Fetal Bovine Serum (FBS) for 48 hours. We normalized the data first to the negative, then the positive control, giving values that represent the percentage of proliferation compared to the positive control. This procedure was done in triplicate to account for non-biological variation (n = 3 + 4 + 4). The percentage of proliferating cells was compared between conditioned media using ANOVA.

#### HUVEC tube formation assays

Previous studies show that HUVECs co-cultured with normal human fibroblasts for 8–14 days form endothelial clusters and complex tube networks that mimic the key phases of *in vivo* angiogenesis^74^. Instead of co-culturing, we modified our experimental set up by transferring conditioned (or control) media to Matrigel seeded HUVECs. HUVECs were seeded on Matrigel in specific chambered 15-well plates with polymer coverslips. We harvested starved HUVECs by trypsin treatment and re-suspended them in a fresh starving medium. We added 500 µL of an experimental conditioned medium to 500 µL of this cell-suspension and seeded 3500 cells/well on Matrigel.

The experiment used seven conditioned media. Conditioned media were collected from one of i) T3M4 culture, ii) NHDF culture, iii) T3M4-NHDF co-culture, iv) T3M4 from ‘extended’ co-culture, or v) NHDF from ‘extended’ co-culture. We also set up two controls using Matrigel seeded HUVECs starving media. The vi) ‘negative control’ was cultured in starving media only, and the vii) ‘positive control’ was supplemented with 1% vascular endothelial growth factor (VEGF) recombinant protein. We incubated cells at 37 °C and quantified angiogenic tube formation four hours post-HUVEC seeding.

Samples were measured using an inverted microscope attached to a digital camera (Leica DM IL LED 11 521 258). We used ImageJ (Wayne Rasband, National Institutes of Health, Bethesda, Maryland, US) and Angiogenesis Analyzer PlugIn (Gilles Carpentier, Faculté des Sciences et Technologie, Université Paris-Est Créteil, Val de Marne, France) to measure the number and length (in pixels) of branches in tube networks. We analysed a central microscopic visual field of the 3500 cells seeded/well at 10x magnification. We normalized each measurement first to the mean of the negative control to establish baseline tube formation in HUVECs (effect size = 0), and then the mean of the positive control to establish a hypothetical effect size of 1. The result is the distance of a given measurement from the mean negative control relative to the distance between control means (i.e. hypothetical effect size of 100%). Experiments and measurements were performed in triplicate to account for non-biological variation.

ANOVA with Tukey’s post-hoc test compared tube formation between conditioned media. We first compared T3M4, NHDF and T3M4-NHDF co-cultures, including also negative and positive controls (n = 3 + 3 + 3 for treatments i, ii, vi and vii). We performed a separate comparison involving the extended co-cultures (treatments iv and v, n = 3 + 6 + 3), singular T3M4 and NHDF (treatments i and ii, n = 6 + 3 + 6), and the controls (treatments vi and vii, n = 6 + 6 + 6). In tube formation assays of extended co-cultures, one branching value of the positive control analysis was censored due to measurement error.

#### Proteome analysis

We used the Proteome Profiler Array (Human Angiogenesis Kit, R&D Systems Europe, Abingdon UK) to identify specific pro-angiogenic factors in the conditioned media. We blocked (array blocking buffer) the Angiogenesis Array membrane for 60 minutes then washed it (1X array wash buffer) before leaving conditioned media to incubate overnight at 4 °C. We pre-treated the media with a 1X lysis buffer provided in the kit. Following incubation, we washed membranes (1X array wash buffer) and incubated streptavidin-HRP solution at room temperature for 30 minutes. We washed membranes three times (1X array wash buffer) and treated them with Lumi Glo and peroxide. We detected signals via Gel Chemiluminescence Imaging - Fusion SL (Vilber Lourmat, Eberhardzell, Baden-Württemberg, Germany). We estimated cytokine concentration by counting spot pixels using Image J software and normalizing means to the negative and positive controls provided by the manufacturer. We conducted one array analysis for single cell-culture media and two for co-culture conditioned media. A factorial ANOVA was used to test cytokine concentration across media.

#### Real-time PCR

We used qPCR analysis to confirm the findings of the proteome analysis on the nuclear level and to identify the cellular source of the secreted CXCL8 and CCL2. We performed the analysis on independently cultured T3M4 and NHDF cells and co-cultures (n = 3 for each of treatments i, ii, iii). We applied the same conditions and cell numbers as per the proteome array. We performed qPCR reactions in triplicate. Relative gene expression was statistically compared using a Welch non-parametric t-test.

We extracted mRNA from single cultures of T3M4 and NHDF cells using primer sets for CXCL8 and CCL2, and generated cDNA for use in qPCR. We extracted total RNA from single cultures seeded at 4 × 10^4^ cells using the RNeasy Plus Mini Kit (Qiagen, Hilden, Germany). 5 µg of total RNA was transcribed using a first-strand cDNA Synthesis Kit (Promega, Mannheim, Germany) following the manufacturer’s instructions. We performed qPCR reactions with 3 µL of cDNA (dilution 1:10) per reaction of SYBR Green, using the Light Cycler 480 SYBR Green I Master (Roche, Mannheim, Germany).

We used gene-specific primers (Supplementary Methods) and quantified relative gene expression using the 2^−∆∆Ct^-method^75^. We normalized the numbers of replication-cycles to gain stable fluorescence (Ct-value) for each gene to the 18 s housekeeping-gene (∆Ct-value). Subsequently, we calculated the relative fold enrichment of each gene in each cell-line by subtracting ∆Ct-values for single-cell-cultures from ∆Ct-values for co-cultures (∆∆Ct-value). Finally, the value of relative expression fold change was calculated as 2^−∆∆Ct^.

#### CXCL8 and CCL2 neutralization assays

We directly examined the effects of CXCL8 and CCL2 on HUVEC tube formation using the same nine replicates from the tube-formation assays: each of the conditioned singular cultures (treatments i and ii), co-culture (treatment iii), control media (treatments vi and vii). We independently mixed the experimental media with a) no additional treatment, b) neutralizing antibodies, c) recombinant proteins, or d) both. Subsequently, we assayed HUVEC tube formation as described above. We compared angiogenesis between treatments and media with two-way ANOVAs, normalizing values as in the tube formation assay. In cases of significant interactions, we performed single effects ANOVAs for the effect of treatment within each non-control media (adjusting the critical p-value to 0.017).

Our neutralizing treatment used antibodies that neutralize CXCL8, CCL2, or the CXCL8 receptors CXCR1 (IL8-Rα) and CXCR2 (IL8-Rβ): 0.5 µg/ml CXCL8, 2.0 µg/ml of CCL2, 2.0 µg/ml CXCR1, 5.0 µg/ml CXCR2. Our recombinant protein treatment used 2.5 ng/ml of CXCL8 recombinant protein and 30 ng/ml of CCL2 recombinant protein.

#### Statistical analysis

Statistical comparisons were performed using Graph PAD Prism 5 (Graphpad Software Inc., La Jolla, CA) and R 3.5.1 (R Core Team, Vienna, Austria). We tested normality with Bartlett’s tests and homogeneity of variance with F-tests. We used a critical p-value of 0.050 unless noted otherwise. Given a target power of 0.80, the sample sizes for the *in vivo* experiment allow us confidence to detect effects greater than 1.09. Our sample sizes for the tube formation and neutralization experiments are reliable for effect sizes greater than 0.55, the proteome analysis 1.13, and qPCR 1.41.

## Supplementary information

Supplementary Material.

## Data Availability

The datasets used and/or analysed in the current study are available from the corresponding author on reasonable request.
