# Supplementary material for: Metastasis-associated fibroblasts promote angiogenesis in metastasized pancreatic cancer via the CXCL8 and the CCL2 axes
Source: Sci Rep. 2020 Mar 25;10:5420. doi: 10.1038/s41598-020-62416-x (PMC7096431; doi:10.1038/s41598-020-62416-x)
Supplement: Supplementary file 1 — Supplementary Material. [file 41598_2020_62416_MOESM1_ESM.pdf]

Metastasis-associated fibroblasts promote angiogenesis in metastasized pancreatic cancer via the CXCL8 and the CCL2 axes.

Thomas M. Pausch, ***Elisa Aue***, *Naita M. Wirsik, Aida Freire Valls, Ying Shen, Praveen Radhakrishnan, Thilo Hackert, Martin Schneider, Thomas Schmidt*

Supplementary Methods

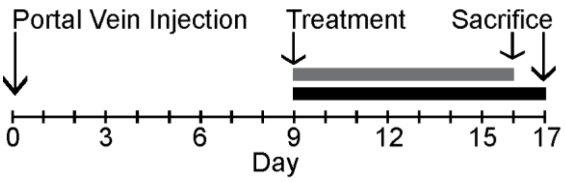

Figure S1: Experimental schedule for inducing hepatic metastases and treating with sunitinib

C57BL/6 female mice induced with PanC02 cells (PBS saline for control) by portal vein injection (day 0), and subsequently treated (from day 9) with daily 40 g/kg sunitinib malate (n = 13) or vehicle solution control (n = 16) before being sacrificed (day 16 or 17). The black bar represents the initial treatment protocol (n = 4 for control, n = 5 for sunitinib treatment), and the grey bar represents the later welfare-compliant protocol.

Table S1: Cell lines and culture ingredients

Cell Line	Culture	Source
Human endothelial cells deriving from umbilical cord veins (HUVECs)	Endopan-3 with Kit containing: 0.1% FGF-2, 0.1% EGF, 0.1% VEGF, 0.1% R3-IGF-1, 0.1% ascorbinic acid, 0.1% heparine, 0.1% gentamicin/amphotericin B, 0.02% hydrocortisone, 10% fetal bovine serum, 1% penicillin/streptomycin	Promocell, Heidelberg, Germany (HUVECs) PAN Biotech, Aidenbach, Germany (medium), Sigma-Aldrich, Darmstadt, Germany (serum, penicillin)
Fast-growing human metastatic pancreatic adenocarcinoma cell line (T3M4)	RPMI-1640 medium, 10% fetal bovine serum, 1% penicillin/streptomycin	R. Metzgar, Duke University, Durham, NC, Px 14-31 (T3M4), Sigma-Aldrich, Darmstadt, Germany (medium), Sigma-Aldrich, Darmstadt, Germany (serum, penicillin)
Normal Human Dermal Fibroblast Cell Line (NHDF)	DMEM, 10% fetal bovine serum and 1% penicillin/streptomycin	Promocell (NHDF), Sigma-Aldrich, Darmstadt, Germany (medium), Sigma-Aldrich, Darmstadt, Germany (serum, penicillin)
Murine pancreatic cancer cells (Panc02)	DMEM, 10% fetal bovine serum and 1% penicillin/streptomycin	Corbett et al. (32), Sigma-Aldrich, Darmstadt, Germany (serum, penicillin)

Chemicals and antibodies

We purchased recombinant human proteins and antibodies from R&D Systems (R&D Systems Europe, Abingdon, UK).

- CXCL8 (catalogue#208-IL)
- CCL2 (catalogue#279-MC)
- Monoclonal neutralizing anti-human CXCL8 (catalogue#MAB208)
- Anti-human CCL2 (catalogue#MAB679)
- Anti-human CXCR1 (catalogue#MAB330)
- Anti-human CXCR2 (catalogue#MAB331)

Gene specific primers for qPCR

- Human CXCL8 forward primer 5’AAG GAA AAC TGG GTG CAG AG’3
- Human CXCL8 reverse primer 5’ATT GCA TCT GGC AAC CCT AC’3
- Human CXCR1 forward primer 5’TGA CTG CAG CTC CTA CTG TTG’3
- Human CXCR1 reverse primer 5’GCA TAC AGG GGC TGT AAT CTT C3’
- Human CXCR2 forward primer 5’CTC CAA TAA CAG CAG GTC ACA G3’
- Human CXCR2 reverse primer 5’AAG ATC TTC ACC TTT CCA GAA ATC3’
- Human CCL2 forward primer 5’ATA GCA GCC ACC TTC ATT CC3’
- Human CCL2 reverse primer 5’AGA TCT CCT TGG CCA CAA TG3’

Sunitinib

Sunitinib is a broad-spectrum inhibitor of several tyrosine kinase receptors (including VEGFR-1-2-3, PDGFR-α,-β, c-KIT, RET and CSF-1R). It is known for its anti-angiogenic effects [1–3]. Sunitinib treatment has recently been FDA approved for the treatment of neuroendocrine pancreatic tumors [4,5].

Supplementary Results

Table S2: Comparison of means ± SD for variables of liver condition in mice from control and sunitinib treatments. Student's t-test was used for all comparisons except liver weight, which used a Welch t-test.

Variable	Control	Sunitinib	t	df	p
Liver weight (g)	2.02 ± 0.26	1.28 ± 0.07	2.82	13.82	0.014
Metastases volume (%)	30.19 ± 5.92	18.75 ± 4.71	1.53	27	0.137
Metastatic load (g)	1.44 ± 0.31	0.31 ± 0.08	3.86	27	< 0.001
Metastases > 1mm	23.38 ± 3.64	13.38 ± 2.55	2.32	27	0.029
Angiogenesis (CD31)	4.27 ± 1.02	4.25 ± 0.82	0.01	24	0.991
Mesenchymal cells (vimentin)	32.88 ± 2.26	26.28 ± 1.52	2.50	27	0.019
MAFs (αSMA)	7.15 ± 0.80	4.82 ± 0.91	1.83	26	0.080
Proliferation (pCNA)	8.12 ± 0.43	12.03 ± 0.48	5.97	26	< 0.001
Lymphocytes (CD45)	8.35 ± 1.04	8.19 ± 0.74	0.13	26	0.899
Macrophages (F4/80)	7.13 ± 1.01	9.38 ± 0.79	1.78	24	0.087

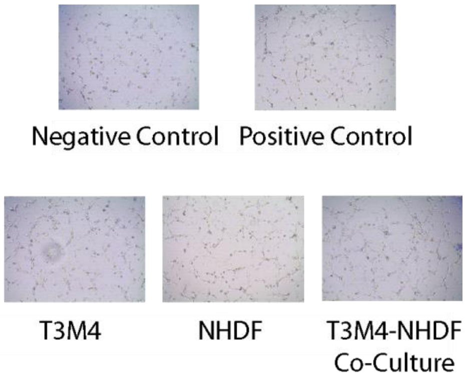

Figure S2: Representative images of HUVECs tube formation

Representative images for HUVECs tube formation in: starving media (negative control), 0.1% VEGF native media (positive control), T3M4 conditioned media, NHDF conditioned media, T3M4-NDHF co-culture conditioned media. HUVECs seeded with Matrigel and cultured for 4 h at 37°C. All images credit by TP and EA.

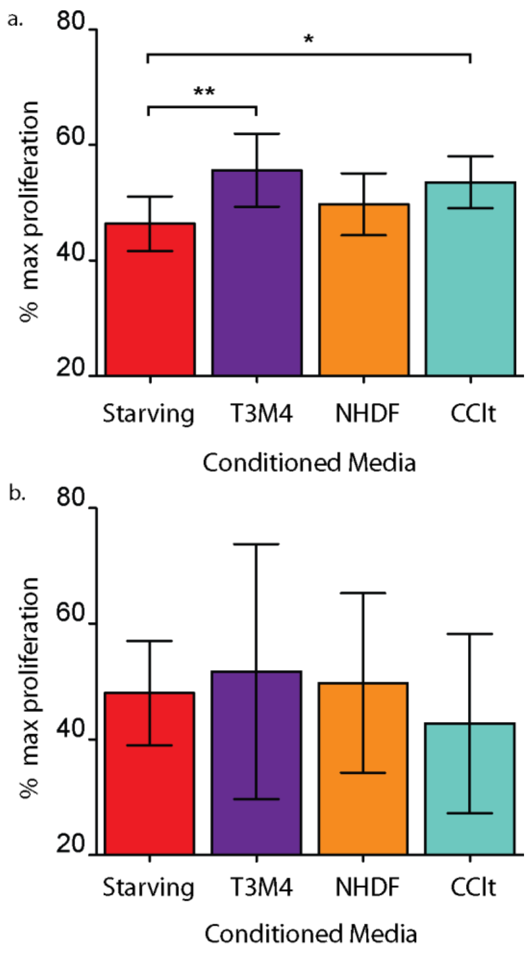

Figure S3: WST-1 assay of cell proliferation

Normalized percentage ± SD of maximum proliferation for (a) NHDF cells and (b) T3M4 cells, 48 hours after incubation in conditioned media. Conditioned media are the negative control (Starving), T3M4, NHDF, and T3M4-NHDF co-culture (CClt). Values are normalized to the difference between negative and positive control means. Experiments were performed in triplicate and analysed with ANOVA, using Tukey's test for pair-wise comparisons: * $p < 0.05$; ** $p < 0.01$; *** $p < 0.001$.

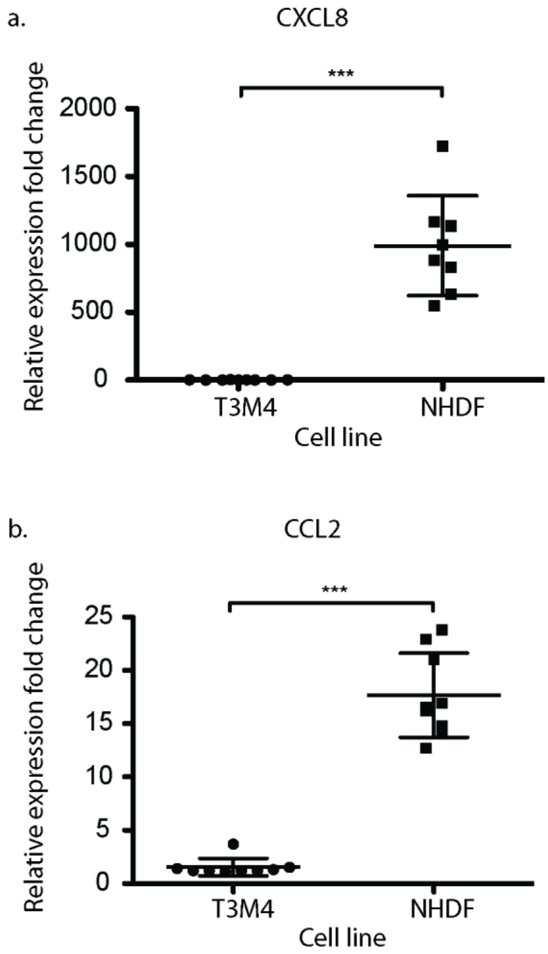

Figure S4: Real-time PCR

Mean relative expression fold change ($2^{\Delta\Delta Ct}$ between mono-culture and co-culture) ± SD detected in genes from T3M4 and NHDF monoculture cells for a) CXCL8 and b) CCL2. Expression is normalized for each gene to the 18s housekeeping-gene (ΔCt-value). Experiments were performed in triplicate. Groups compared using Students t-test: * $p < 0.05$; ** $p < 0.01$; *** $p < 0.001$.

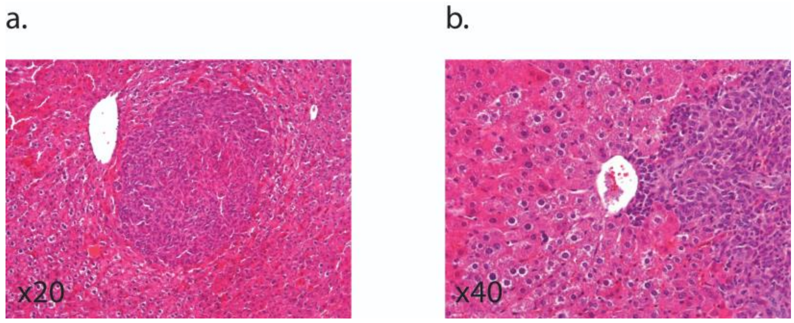

Figure S5: Representative images of immunohistochemical slides with hemalaun stainings and eosin counterstainings.

Images at (a) x20 magnification and (b) x40 magnification. All images credit by TP and EA.

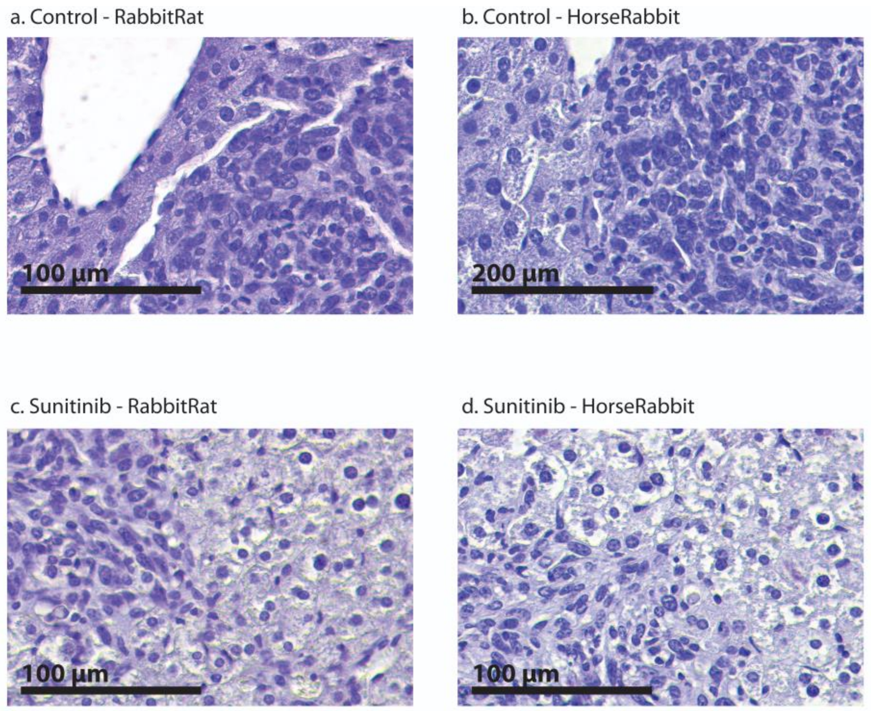

Figure S6: Representative images of immunohistochemical slides for negative sample controls.

Slides for (a, b) untreated and (c, d) sunitinib treated mice, using only secondary (a, c) Rabbit-Rat and (b, d) Horse-Rabbit antibodies. All images credit by TP and EA.

Tables S3-S6: Two-Way ANOVAs for the effects of antibody neutralization and experimental media on the normalized number of tube branches and their total length. Antibodies are used to neutralize proteins CCL2 (Table S3), CXCL8 (Table S4), and CXCL8 receptors CXCR1 (Table S5), CXCR2 (Table S6). Values are normalized to the mean distance between negative and positive controls.

Table S3:

	CCL2			
	Number		Length	
**Treatment (df)**	**F**	**p**	**F**	**p**
Antibody x Media (4,80)	8.09	< 0.001	10.46	< 0.001
Antibody (1, 80)	7.38	0.008	13.22	< 0.001
Media (4, 80)	27.32	< 0.001	26.80	< 0.001

Table S4:

	CXCL8			
	Number		Length	
**Treatment (df)**	**F**	**p**	**F**	**p**
Antibody x Media (4,80)	6.11	< 0.001	6.76	< 0.001
Antibody (1, 80)	0.48	0.493	5.96	0.017
Media (4, 80)	20.02	< 0.001	22.94	< 0.001

Table S5:

	CXCR1			
	Number		Length	
**Treatment (df)**	**F**	**p**	**F**	**p**
Antibody x Media (4,80)	12.37	< 0.001	16.08	< 0.001
Antibody (1, 80)	5.22	0.025	6.08	0.016
Media (4, 80)	27.92	< 0.001	26.46	< 0.001

Table S6:

	CXCR2			
	Number		Length	
**Treatment (df)**	**F**	**p**	**F**	**p**
Antibody x Media (4,80)	10.85	< 0.001	13.92	< 0.001
Antibody (1, 80)	13.25	< 0.001	22.86	< 0.001
Media (4, 80)	18.65	< 0.001	19.37	< 0.001

Table S7-S10: One-Way ANOVAs for the effects of antibody neutralization on the normalized number of tube branches and their total length, within each experimental medium. Antibodies are used to neutralize proteins CCL2 (Table S7), CXCL8 (Table S8), and CXCL8 receptors CXCR1 (Table S9), CXCR2 (Table S10). Tests are against a critical p-value of 0.05/3 = 0.017. Values are normalized to the mean distance between negative and positive controls.

**Table S7:**	**CCL2**			
	**Number**		**Length**	
**Media**	**$F_{1,16}$**	**p**	**$F_{1,16}$**	**p**
T3M4	0.02	0.879	0.38	0.548
NHDF	0.18	0.679	0.20	0.658
Co-Culture	36.81	< 0.001	44.29	< 0.001

**Table S8:**	**CXCL8**			
	**Number**		**Length**	
**Media**	**$F_{1,16}$**	**p**	**$F_{1,16}$**	**p**
T3M4	1.39	0.256	0.23	0.635
NHDF	1.20	0.290	0.06	0.807
Co-Culture	8.92	0.009	13.88	0.002

**Table S9:**	**CXCR1**			
	**Number**		**Length**	
**Media**	**$F_{1,16}$**	**p**	**$F_{1,16}$**	**p**
T3M4	0.01	0.907	0.52	0.481
NHDF	0.94	0.346	1.67	0.215
Co-Culture	37.22	< 0.001	55.88	< 0.001

**Table S10:**	**CXCR2**			
	**Number**		**Length**	
**Media**	**$F_{1,16}$**	**p**	**$F_{1,16}$**	**p**
T3M4	1.41	0.252	1.51	0.237
NHDF	0.50	0.490	0.01	0.908
Co-Culture	60.27	< 0.001	78.60	< 0.001

Tables S11-S12: Two-Way ANOVAs for the effects of recombinant proteins and experimental media on the normalized number of tube branches and their total length. Added recombinant proteins are CCL2 (Table S11) and CXCL8 (Table S12). Values are normalized to the mean distance between negative and positive controls.

Table S11: CCL2

	Number		Length	
**Treatment (df)**	**F**	**p**	**F**	**p**
Protein x Media (4,80)	1.88	0.123	3.92	0.006
Protein (1, 80)	20.17	< 0.001	43.84	< 0.001
Media (4, 80)	8.35	< 0.001	10.22	< 0.001

Table S12: CXCL8

	Number		Length	
**Treatment (df)**	**F**	**p**	**F**	**p**
Protein x Media (4,80)	5.28	< 0.001	6.89	< 0.001
Protein (1, 80)	15.74	< 0.001	57.08	< 0.001
Media (4, 80)	6.96	< 0.001	18.72	< 0.001

Tables S13-S14: One-Way ANOVAs for the effects of recombinant proteins on the normalized number of tube branches and their total length, within each experimental medium. Added recombinant proteins are CCL2 (Table S13) and CXCL8 (Table S14). Tests are against a critical p-value of 0.05/3 = 0.017. Values are normalized to the mean distance between negative and positive controls.

Table S13: CCL2

	Number		Length	
**Media**	$F_{1,16}$	**p**	$F_{1,16}$	**p**
T3M4	7.54	0.014	15.67	0.001
NHDF	5.88	0.028	8.80	0.009
Co-Culture	0.13	0.723	1.33	0.266

Table S14: CXCL8

	Number		Length	
**Media**	$F_{1,16}$	**p**	$F_{1,16}$	**p**
T3M4	7.15	0.017	20.97	< 0.001
NHDF	1.38	0.257	8.96	0.009
Co-Culture	1.31	0.269	1.45	0.246

Tables S15-S16: Two-Way ANOVAs for the effects of experimental media and neutralizing recombinant proteins with antibodies on the normalized number of tube branches and their total length. Antibodies are used to neutralize recombinant proteins CCL2 (Table S15), CXCL8 (Table S16). Values are normalized to the mean distance between negative and positive controls.

Table S15:

	CCL2			
	Number		Length	
**Treatment (df)**	**F**	**p**	**F**	**p**
Antibody x Media (4,80)	0.59	0.671	0.25	0.909
Antibody (1, 80)	10.71	0.002	27.71	< 0.001
Media (4, 80)	4.14	0.004	2.94	0.026

Table S16:

	CXCL8			
	Number		Length	
**Treatment (df)**	**F**	**p**	**F**	**p**
Antibody x Media (4,80)	0.635	0.639	0.586	0.674
Antibody (1, 80)	23.29	< 0.001	45.26	< 0.001
Media (4, 80)	3.55	0.010	4.09	0.005

Supplementary Discussion

Neutralization Experiments

The results of our antibody neutralization experiments require further consideration not provided in the main manuscript. Specifically, we found that the addition of recombinant proteins to co-cultured media did not lead to significantly greater angiogenesis, as in mono-cultures (Figure 6b). We believe that this is due to a saturation effect, as co-cultured media may already have intrinsic target proteins binding to receptors at a high rate, so the addition of recombinant proteins has a small effect expression rate.

Adding antibodies to co-cultured media with recombinant proteins also did not result in a significant inhibition of angiogenesis, as seen in mono-cultures (Figure 6c). This may similarly be due to the high concentration of intrinsic proteins. That is, angiogenesis is significantly reduced in co-culture without recombinant proteins (Figure 6a), implying that antibodies are effective at lower protein concentrations. Additionally, as mentioned, recombinant proteins are ineffective at higher concentrations (Figure 6b), possibly due to a saturation effect. But, even so, Bonferroni post-hoc tests suggest the antibody effect is smaller in co-culture compared to other media. Thus, the lack of a strong negative effect of antibodies in co-culture with recombinant proteins may be due to the higher protein concentration in co-cultured media compared to mono-cultures. That being said, it is important to note that our analysis found a significant negative main effect of antibodies across all media (i.e. there was no media * antibody interaction), and it is clear from the figures that angiogenesis was lower after the addition of antibodies.

Supplementary References

1. Mendel, D. B. *et al.* In vivo antitumor activity of SU11248, a novel tyrosine kinase inhibitor targeting vascular endothelial growth factor and platelet-derived growth factor receptors: determination of a pharmacokinetic/pharmacodynamic relationship. *Clin Cancer Res* **9**, 327–337 (2003).

2. Abrams, T. J., Lee, L. B., Murray, L. J., Pryer, N. K. & Cherrington, J. M. SU11248 inhibits KIT and platelet-derived growth factor receptor beta in preclinical models of human small cell lung cancer. *Mol Cancer Ther* **2**, 471–478 (2003).

3. O'Farrell, A. M. *et al.* SU11248 is a novel FLT3 tyrosine kinase inhibitor with potent activity in vitro and in vivo. *Blood* **101**, 3597–3605 (2003).

4. Blumenthal, G. M. *et al.* FDA approval summary: sunitinib for the treatment of progressive well-differentiated locally advanced or metastatic pancreatic neuroendocrine tumors. *Oncologist* **17**, 1108–1113 (2012).

5. Vinik, A. I. & Raymond, E. Pancreatic neuroendocrine tumors: approach to treatment with focus on sunitinib. *Ther. Adv Gastroenterol* **6**, 396–411 (2013).
